# Comparison of self- versus clinician-administered GAIN-Q3 MI

**DOI:** 10.1186/1940-0640-10-S1-A57

**Published:** 2015-02-20

**Authors:** Brian Rush, Felicia Tan, Nancy Chau

**Affiliations:** 1 Centre for Addiction and Mental Health, Toronto, Ontario, Canada, M5S 2S1; 2 Department of Psychology, University of Toronto, Toronto, Ontario, Canada, M5S 2J7

## Background

Substance abuse services treatment providers are challenged in making decisions regarding evidence-based assessment tools and processes, given the many options available in terms of length/coverage and administration options, including clinician versus self-administration. In Ontario, Canada, we have pilot-tested an assessment tool designed for clinician administration (the GAIN Q3-MI), with the aim being to replace a suite of tools suitable for self-completion. Feedback from the agencies involved in the pilot testing raised concerns about the impact on agency waiting lists if the treatment network was to move to a clinician-administered option. The present project was a small-scale experiment of face-to-face versus self-administration of the GAIN tool, intended to assess differences in data quality and completeness, staff feedback on pros and cons, and client feedback on acceptability of the alternative methods of administration.

## Method

Thirty-eight eligible adult clients presenting at a community addiction agency for assessment and treatment were randomly assigned to either self-complete the GAIN-Q3 MI on a laptop (n = 19) or have a clinician administer the GAIN-Q3 MI (n = 19). Clients were then asked to complete a survey on their assessment experience. The final sample for analysis was 35 participants; 18 self-administered and 17 clinician-administered. Data from the auto-generated validity report, which summarizes data inconsistencies identified during the test administration, and time taken to complete the assessment were collected.

## Results

There was a small statistically significant difference between groups in errors identified—the self-administered  = 3.61 (SD = 2.30) and the clinician-administered  = 2.21 (SD = 1.58). These differences between groups were particularly salient in the risk behaviors domain (*t*(35) = –2.77; *p* = .009). No significant difference was found between groups in terms of completion time (*t*(34) = –1.88; *p* = 0.07). The majority of clients in each group found their method of administration acceptable (82.4% of self-administered; 87.5% of clinician-administered). Interestingly, a client with hearing impairment commented positively on the self-administered version since an interpreter was not needed. Feedback from staff was positive on the self-administration option.

## Conclusions

The current study shows that the self-administration option provides an effective means for clients to self-complete the GAIN-Q3 MI with the use of technology. To facilitate the process of self-administration, it is recommended that a separate self-administered version of the GAIN-Q3 MI be produced and clinician support provided to facilitate interpretation when needed, especially for reporting of risk behaviors.

